# Clinicopathologic Characteristics of Grade 2/3 Meningiomas: A Perspective on the Role of Next-Generation Sequencing

**DOI:** 10.3389/fonc.2022.885155

**Published:** 2022-06-13

**Authors:** Junhyung Kim, Kihwan Hwang, Hyun Jung Kwon, Ji Eun Lee, Kyu Sang Lee, Gheeyoung Choe, Jung Ho Han, Chae-Yong Kim

**Affiliations:** ^1^ Department of Neurosurgery, Seoul National University Bundang Hospital, Seoul National University College of Medicine, Seongnam, South Korea; ^2^ Department of Pathology, Seoul National University Bundang Hospital, Seoul National University College of Medicine, Seongnam, South Korea

**Keywords:** meningioma, atypical meningioma, anaplastic meningioma, next-generation sequencing, *TERT* promoter mutation

## Abstract

**Background:**

Grade 2/3 meningiomas have locally aggressive behaviors often requiring additional treatment plans after surgical resection. Herein, we explored the clinical significance of next-generation sequencing (NGS) in characterizing the molecular profiles of high-grade meningiomas.

**Methods:**

Patients with intracranial meningioma who underwent surgical resection in a single institution were retrospectively reviewed. Clinicopathologic relevance was evaluated using recurrence-free survival (RFS) as an outcome measure. NGS for the targeted gene regions was performed in 40 participants.

**Results:**

Among the 713 individuals in the study population, 143 cases (20.1%) were identified as having grade 2 or 3 meningiomas with a significantly lower female predominance. While the difference in RFS between grade 2 and 3 meningiomas was insignificant, a few conventional grade 2 cases, but with *TERT* promoter hotspot mutation, were highly progressive and refractory to the treatment. From the NGS study, recurrent mutations in *TRAF* and *AKT1* were identified with a higher prevalence (17.5% and 12.5%, respectively) compared with grade 2/3 meningiomas reported in previous literature. However, their relations to other histopathologic properties or clinical factors were rarely observed.

**Conclusions:**

Grade 2/3 meningiomas show a broad spectrum of molecular profiles, as they have heterogeneous histologic characteristics.

## Introduction

Meningioma is a single pathologic entity of benign extra-axial tumors in the central nervous system (CNS) with a broad morphological spectrum reflected in various histopathologic subtypes (1). Although exhibiting a mostly benign nature, up to 25% of cases have shown to have aggressive behaviors, as classified as grade 2 or 3 (2, 3). The primary mode of treatment is surgery; however, due to anatomical obstacles or some other circumstances, complete tumor resection is often unachievable. The tumor characteristics, together with the surgical outcome, have been considered an important prognostic factor associated with local recurrence, and additional treatment plans are always considered for grade 2/3 meningiomas.

Recently, numerous studies have suggested molecular signatures representing aggressive tumor characteristics and several specific biomarkers, i.e., *TERT* promoter mutation and *CDKN2A/B* homozygous deletion, which have been added to the updated diagnostic criteria for anaplastic meningiomas (1, 4). This, in an addition to the rapid adoption of next-generation sequencing (NGS) beside clinical practice, has increased the interests in genomic characterization of specific meningioma subtypes (3). Thus, we explored the clinical significance of molecular diagnostic studies in grade 2/3 meningiomas. We also validated previously published recurrent mutations and their clinicopathologic relevance in our cohort with the NGS study.

## Materials and Methods

### Study Population

This study was intended for patients with intracranial meningioma. The study population was retrospectively selected from the institutional tumor registry. Patients who had agreed with biobanking and genetic testing of tumor tissue for a scientific purpose have enrolled under the approval of the institutional review board. Patients were not compensated for their participation, and each participant provided written informed consent. All experiments were performed following the relevant guidelines and regulations.

### Tissue Acquisition and Histopathologic Diagnosis

Formalin-fixed paraffin-embedded (FFPE) tissue specimens were obtained after surgical resection. Two senior pathologists (K.S.L. and G.C.) independently reviewed the hematoxylin and eosin (H&E)-stained slides and confirmed the diagnosis according to the fourth edition of the World Health Organization classification of tumors of the CNS (WHO CNS) to identify the histological subtypes of meningiomas.

Immunostaining was performed for a single representative sample block showing specific histomorphologic characteristics from each case. Sections were transferred to poly-L-lysine coated adhesive slides, dried, deparaffinized, and rehydrated. The slides were subsequently subjected to heat-induced antigen retrieval. The following antibodies were used according to the manufacturer’s instruction: c-MET (prediluted, rabbit monoclonal antibody, Ventana, Tucson, AZ, USA), EGFR (1:150, mouse monoclonal antibody, Dako, Camarillo, CA, USA), p16 (prediluted, mouse monoclonal antibody, Ventana, Tucson, AZ, USA), and Ki-67 (1:50, mouse monoclonal antibody, Dako, Carpinteria, CA, USA). The sections were incubated with appropriate reagents from the Dako REAL EnVision Detection System (Dako, Glostrup, Denmark) and were counterstained with Mayer’s hematoxylin. Each case was categorized as either positive — when it showed moderate-to-strong cytoplasmic and/or membranous positivity in tumor cells — or negative, as controls without antibodies. For p16 staining, loss of expression was recorded if the tumor cells showed complete negative, while partial expression was considered positive.

### *TERT* Promoter Mutation Analysis by Pyrosequencing

A sample FFPE block with the most representative morphologic characteristics was selected from each case, and tumor areas were manually microdissected from the unstained slides. DNA was extracted using the Maxwell 16 FFPE Purification Kit (Promega, Madison, WI, USA) according to the manufacturer’s instructions. The *TERT* promoter region covering the hotspot mutations C228T (g.1,295,228C>T in GRCh37) and C250T (g.1,295,250C>T) were amplified by polymerase chain reaction (PCR) using the HotStarTaq Plus Master Mix Kit (Qiagen, Valencia, CA, USA) and primers forward 5’-GTCCTGCCCCTTCACCTT-3’ and reverse 5’-CAGCGCTGCCTGAAACTC-3’, which was run on Verti 96-well Thermal Cycler (Applied Biosystems, Waltham, MA, USA) with the following conditions: 96°C for 5 min; 44 cycles with 96°C for 30 s, 64°C for 30 s and 72°C for 1 min; 72°C for 10 min and 4°C for hold. The quality of PCR products was confirmed by gel electrophoresis on 2% agarose gel. Pyrosequencing of the *TERT* promoter mutations C228T and C250T was carried out on the PyroMark Q24-MDX system (Qiagen, Valencia, CA, USA) with a sequencing primer 5’-ACCCCGCCCCGTCCCGACCCC-3’, based on the manufacturer’s instructions. TERT promoter mutation was considered positive if T allele frequency at the mutation sites was ≥10%.

### Genomic Characterization by Next-Generation Sequencing

DNA was extracted from the FFPE tissue sample using the QIAamp DNA FFPE Tissue Kit (Qiagen, Valencia, CA, USA) according to the manufacturer’s instructions. The extracted DNA was quantitated using a Quantus fluorometer (Promega, Madison, WI, USA) and Agilent 4200 TapeStation system (Agilent Technologies, Santa Clara, CA, USA). DNA was sheared into fragments with a mean peak size of approximately 180 bp to 200 bp, using Adaptive Focused Acoustics (Covaris, Woburn, MA, USA). The paired-end libraries were prepared with a SureSelectXT HS Target Enrichment System kit (Agilent Technologies, Santa Clara, CA, USA), based on the manufacturer’s instruction, using commercially available targeted panels that cover *NF2*, *AKT1*, *TRAF7*, *KLF4*, *SMO*, *PIK3CA*, *SMARCB1*, and other cancer-related genes (**Supplementary Table 1**). The quality of the DNA library was evaluated using a Bioanalyzer 2100 (Agilent Technologies, San Francisco, CA, USA) and quantified using a Qubit dsDNA HS Assay kit and a Qubit 2.0 fluorometer (Life Technologies, Carlsbad, CA, USA). The libraries were paired-end sequenced (2 × 150 bp) using a NextSeq 550Dx (Illumina, San Diego, CA, USA) or a HiSeq 2500 (Illumina, San Diego, CA, USA).

Sequencing data quality assessment and trimming were performed using FastQC v0.11.8 (RRID : SCR_014583). The sequence reads were aligned to the reference genome GRCh37 (hg19) using BWA v0.7.15 (RRID : SCR_010910), Picard v2.16.0 (RRID : SCR_006525), and Samtools v1.3.1 (RRID : SCR_002105). Single nucleotide variants (SNVs) and indels were identified using GATK v4.1.4.0 (RRID : SCR_001876), where a threshold of at least 10 reads and an allelic frequency of ≥10% were used for variant calling. The sequencing variants were annotated using SnpEff & SnpSift v5.1 (RRID : SCR_005191/SCR_015624). The variants found in the population databases, the Exome Aggregation Consortium (RRID : SCR_004068), and the Genome Aggregation Database (RRID : SCR_014964), were filtered out to demonstrate somatic mutations minimizing germline contamination. All variants were interpreted based on the Association for Molecular Pathology (AMP) standards and guidelines (5).

### Clinical Evaluation

All patients received regular follow-up after initial treatment. Magnetic resonance images (MRIs) were performed within 48 hours after surgery, and the extent of resection was evaluated by neurosurgeons. For patients with visible residual tumors, adjuvant stereotactic radiosurgery or radiotherapy was usually performed within three to six months from surgery. Patients with grade 2 or 3 meningiomas were referred to the institution’s multidisciplinary neuro-oncology outpatient clinic, where a team of neurosurgeon, neuroradiologist, pathologist, medical oncologist, and radiation oncologist participated in a discussion to select the most optimal therapeutic plan for each patient.

Patients were usually followed up every six months for the first two years and then annually thereafter. The recurrence-free survival (RFS) was calculated from the date of surgical resection to the first recurrence of meningioma requiring further therapeutic plans. Evidence of local tumor recurrence was provided by MRI showing meningioma in a location contiguous with the previous operation site.

### Statistical Consideration

For descriptive statistics, the frequency with percentages was provided for categorical data and median with interquartile ranges for continuous data. Differences between grades 1 and 2/3 meningiomas were compared using the Chi-square test for categorical variables and the t-test for continuous variables. The statistical correlations between genomic features and clinicopathological parameters were evaluated using the Fisher’s exact test. Differences in RFS between distinct subgroups were assessed by the log-rank test. The prevalence of known recurrent variants in this study was compared to the pooled data from external cohorts using the Chi-squared test. Each result from statistical tests was considered statistically significant if a two-tailed p-value was less than 0.05. All statistical analyses were performed using R v3.6.3 (RRID : SCR_001905).

## Results

### Baseline Characteristics

**Figure 1:**
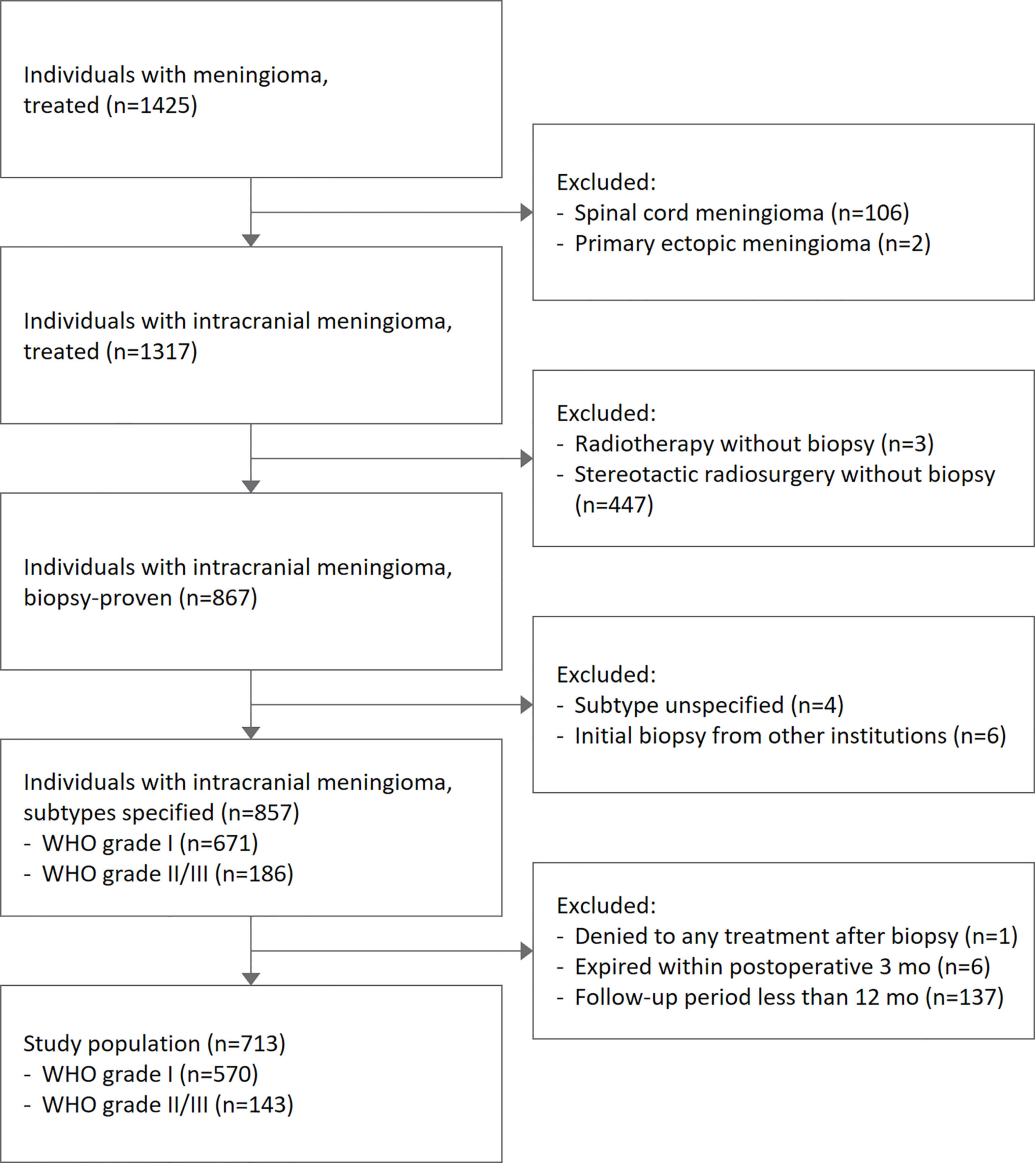
Selection of study population.

A total of 713 cases with intracranial meningiomas who received surgical treatment between 2003 and 2020 were reviewed (**Figure 1**). The median age of the study population was 56 (47-65) years, and females made up 71.6% of the population. Grade 2/3 meningiomas were identified in 143 (20.1%) cases. Patients with grade 2/3 meningiomas tended to have a lower female predominance compared with those with grade 1 tumors (**Table 1**): male, 44.8% vs. 24.3% (p < 0.001). There was no significant association between grades and the location of tumors.

**Table 1:** Baseline characteristics of the study population.

	Study cohort (n=713)		Sample (n=40)
	Grade 1 (n=570)	Grade 2/3 (n=143)	
**Age (yr)**	56 [47, 64]	57 [47, 67]	55 [48, 66]
≥65	136 (24)	45 (31)	11 (28)
**Sex**			
Male	138 (24)	64 (45)	16 (40)
**Location**			
Convexity	164 (30)	46 (32)	22 (55)
Parasagittal/falx	95 (17)	43 (30)	12 (30)
Skull-based^†^	244 (43)	45 (31)	4 (10)
Intraventricular	11 (2)	8 (6)	2 (5)
Miscellaneous	51 (9)	1 (1)	.
**Etiology**			
Sporadic	568 (100)	139 (97)	40 (100)
NF2	2 (0)	3 (2)	.
Radiation	.	1 (1)	.
**Prior treatment**			
Untreated	561 (99)	128 (90)	37 (92)
Treated	9 (1)	15 (10)	3 (8)
**Treatment type**			
Resection only	491 (86)	68 (48)	13 (33)
Resection + SRS	76 (13)	22 (15)	8 (20)
Resection + RT	3 (1)	53 (37)	19 (48)
**Pathology**			
Meningothelial	232 (41)	.	.
Fibrous	95 (17)	.	.
Transitional	152 (27)	.	.
Psammomatous	16 (3)	.	.
Angiomatous	44 (8)	.	.
Microcystic	19 (3)	.	.
Secretory	3 (1)	.	.
LPR	1 (0)	.	.
Metaplastic	8 (1)	.	.
Atypical	.	123 (86)	35 (88)
Chordoid	.	5 (3)	.
Clear cell	.	.	.
Anaplastic	.	8 (6)	2 (5)
Papillary	.	4 (3)	1 (3)
Rhabdoid	.	3 (2)	2 (5)

Values are median [IQR] or number (percent). ^†^Skull-based includes olfactory groove, planum sphenoidale, tuberculum sellae, sphenoid wing, cavernous sinus, petroclival, petrosal, and foramen magnum lesions. NF2, neurofibromatosis type 2; LPR, lymphoplasmocyte-rich; SRS, stereotactic radiosurgery; RT, radiotherapy.

A sample of 40 patients with grade 2/3 meningiomas participated in the targeted NGS panel study, whose tissue specimens were acquired between 2012 and 2019. The baseline characteristics of the sample and the study population were evenly distributed. Most candidates provided the tissue specimens from the initial surgery, except three patients with recurrent meningiomas who had a history of prior surgery and/or stereotactic radiosurgery. The candidates had no past medical history of underlying malignancies at the time of tissue acquisition, except one patient who had extracranial metastasis and received systemic chemotherapy with hydroxyurea before tumor sampling.

### Clinical Outcome and Molecular Diagnosis

**Figure 2:**
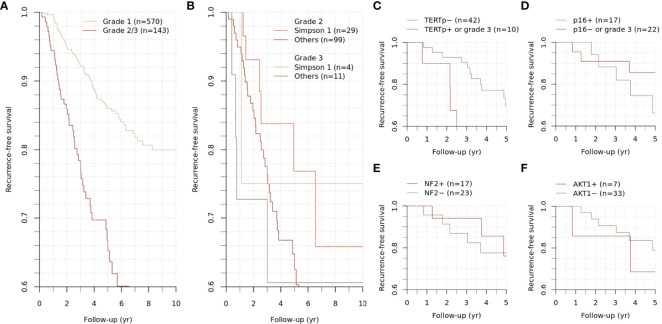
Recurrence-free survival of grade 2/3 meningiomas. Differential recurrence-free survival was observed between grade 1 and grade 2/3 meningiomas **(A)**. The survival difference between grades 2 and 3 was insignificant **(B)**. The subgroups of grade 2/3 meningiomas were stratified according to the mutational status of specific genes **(C–F)**.

The study population was followed up for 59.1 (30.0-98.0) months. During the follow-up period, 59 cases of grade 1 meningiomas and 40 cases of grade 2/3 meningiomas had an event of recurrence, showing distinct outcomes (p < 0.001) (**Figure 2A**). Five-year recurrence-free survival (RFS) estimates were 86.0 [95% CI, 82.7-89.5%] and 66.8% [95% CI, 58.5-76.2%] for grade 1 and 2/3 meningiomas, respectively.

For grade 2/3 meningiomas, Simpson grade I resection was achieved in 33 (23.1%) cases, followed by 44 (30.8%) cases for grade II, 19 (13.3%) cases for grade III, 43 (30.1%) cases for grade IV, and 4 (2.8%) cases for grade V resection. While the survival difference between grades 2 and 3 was insignificant, the achievement of Simpson grade I resection significantly influenced the clinical outcome in grade 2 meningiomas (p = 0.005) (**Figure 2B**). Other treatment factors regarding adjuvant stereotactic radiosurgery or radiotherapy did not significantly change the outcome.

In the sample population, local recurrence has been developed in 10 patients during the follow-up period of 68.5 (55.8-97.3) months. Simpson grade I resection was achieved in 10 (25.0%) cases. Those patients with recurrence received additional treatment with stereotactic radiosurgery, and two of them underwent reoperation for surgical resection of the recurred tumor. The five-year RFS was 65.4% for a subset of participants whose long-term follow-up data were available, representing the grade 2/3 meningiomas in the study cohort.

**Figure 3:**
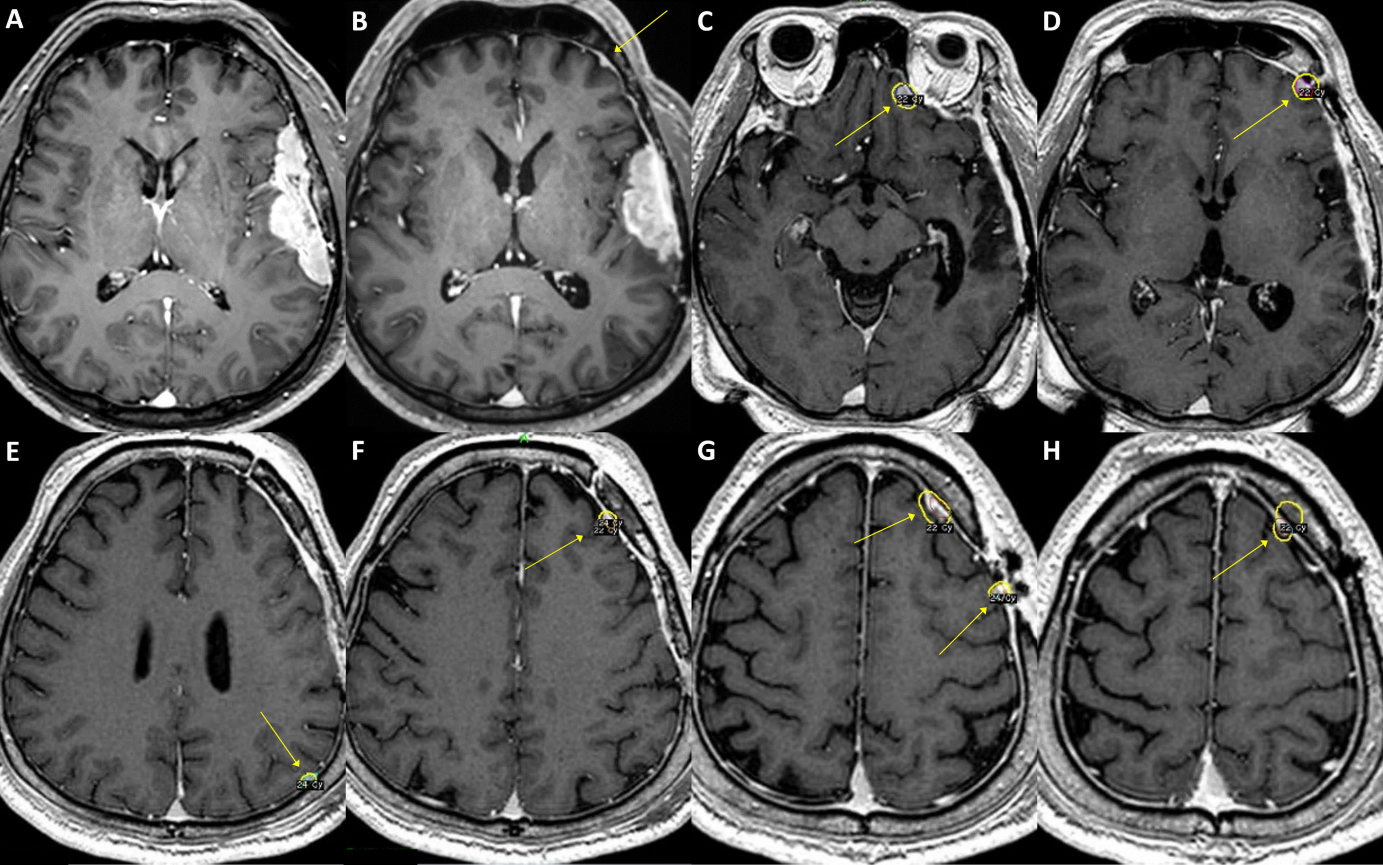
A case of convexity meningioma with *TERT* promoter mutation. A 52-year-old male presented with an en plaque meningioma in the left temporal convexity **(A)**. The tumor was partially resected during the initial surgery, and the residual superior temporal mass, severely adhered to the brain parenchyma and the vascular structures in the Sylvian fissure, was further treated by adjuvant stereotactic radiosurgery. However, the tumor regrew **(B)**, and another operation was performed after two years from the initial treatment. The specimens from the first and second operations both presented C250T *TERT* promoter mutation. Although the primary lesion was successfully removed, a new, tiny lesion at the left frontal convexity (**B**, arrow) had been rapidly grown at six-month follow-up, in addition to other multiple distant lesions (**C–H**, arrows). The patient underwent stereotactic radiosurgery six times over the next five years, one more reoperation at the primary location, and then another two times of stereotactic radiosurgery for the refractory tumors. In 9 years from the initial treatment, the leptomeningeal spread of the tumor cells was confirmed by cerebrospinal fluid examination, and the patient received a ventriculoperitoneal shunt operation for hydrocephalus.

**Figure 4:**
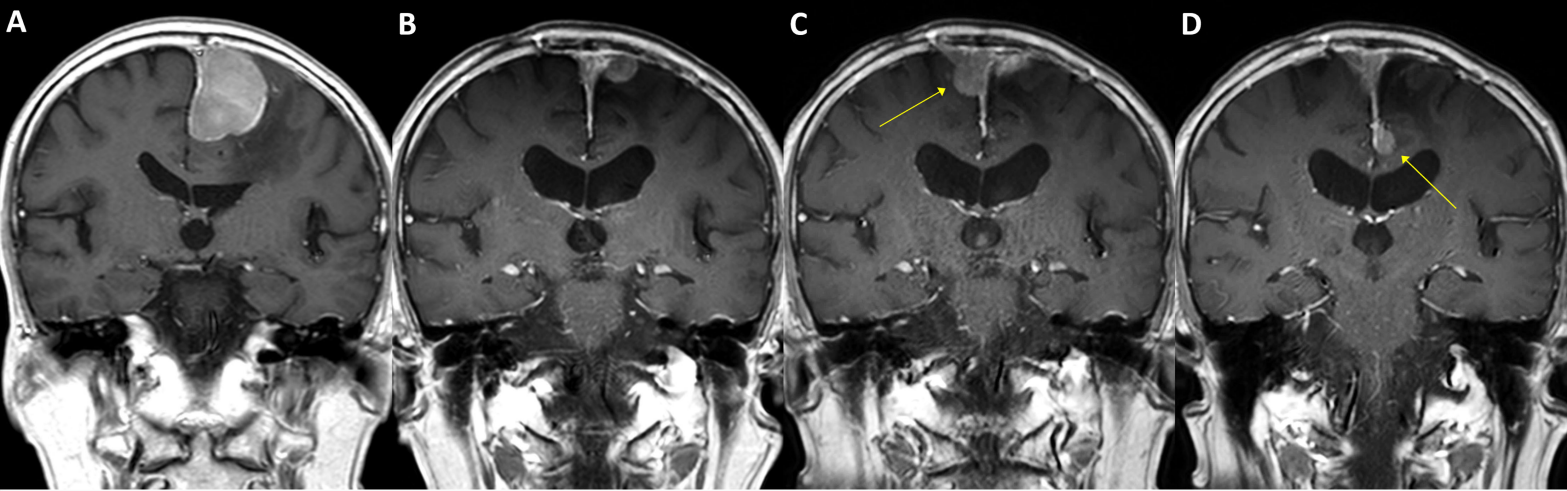
A case of parasagittal meningioma with *TERT* promoter mutation. A 78-year-old male with a 4.5 cm-sized left parasagittal meningioma **(A)** underwent surgery achieving subtotal resection, followed by adjuvant radiotherapy. In two years, the tumor recurred at the primary location **(B)** and was treated by secondary radiosurgery. In the following year, two new lesions as well as the progressed primary lesion were detected (**C, D**, arrows) and retreated by radiosurgery. The patient had suffered from impaired gait function and expired due to pneumonia after having a femur neck fracture on the sixth year from the initial treatment. The specimen acquired from the initial surgery has presented C228T *TERT* promoter mutation.

**Figure 5:**
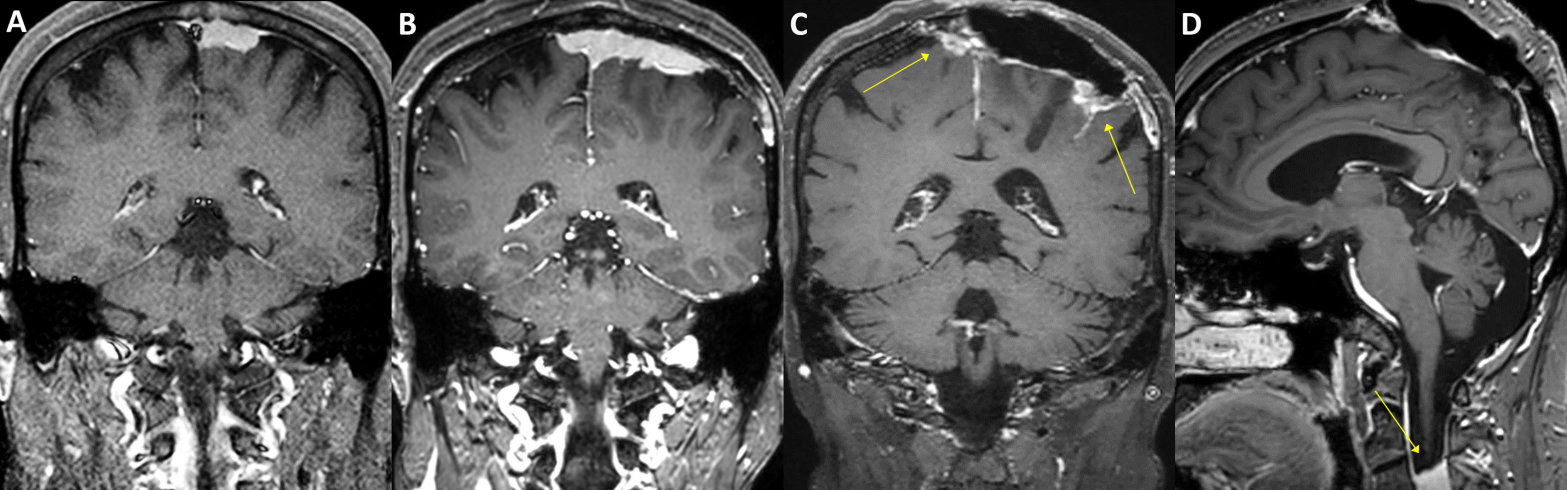
A case of parasagittal meningioma with *TERT* promoter mutation. A 47-year-old female with a 2.5 cm-sized left parasagittal meningioma **(A)** underwent stereotactic radiosurgery. In seven years, the tumor, however, progressed **(B)** and was treated by partial resection and adjuvant radiotherapy. The tumor was refractory to the treatments and required another operation and three-stage radiosurgery after two years. The tumor still progressed in the following year (**C**, arrows) and was further treated with radiosurgery three times. One year after, a total of 11 years from the initial treatment, a new rapidly growing lesion was found in the second cervical spine level (**D**, arrow). Multiple pulmonary metastases were also found in the positron-emission tomography scan. The patient received laminectomy and tumor removal for the distant lesion, and the specimen presented C228T *TERT* promoter mutation.

To evaluate the clinicopathologic relevance regarding the molecular diagnostic criteria in WHO CNS 5, we assessed the *TERT* promoter and *CDKN2A/B* status of meningioma. The *TERT* promoter hotspot mutation PCR was available for 52 cases (40 samples and additional 12 cases) with grade 2/3 meningiomas. Positive results were identified in three cases; these were conventionally diagnosed as atypical meningiomas with high mitotic activity, and all recurred during the follow-up period and highly refractory to the selected treatments (**Figures 3**–**5**). Interestingly, two of them had distant metastasis with rapid progression. Those formerly diagnosed as grade 3 meningiomas (7 cases) did not present a positive *TERT* promoter mutation. The molecularly integrated classifying group with conventional grade 3 and positive *TERT* promoter mutation showed a poorer outcome than the others (**Figure 2C**).

We also assessed the immunohistochemistry of CDKN2A (p16) that homozygotic deletion of the corresponding gene would be represented by loss of expression. Among 40 sample participants, 17 patients with grade 2 meningiomas were reclassified into a higher grade, as they presented negative p16 staining. However, the immunohistochemical status of CDKN2A did not show a significant difference in RFS (**Figure 2D**).

### Mutational Profiling of Grade 2/3 Meningiomas

**Figure 6:**
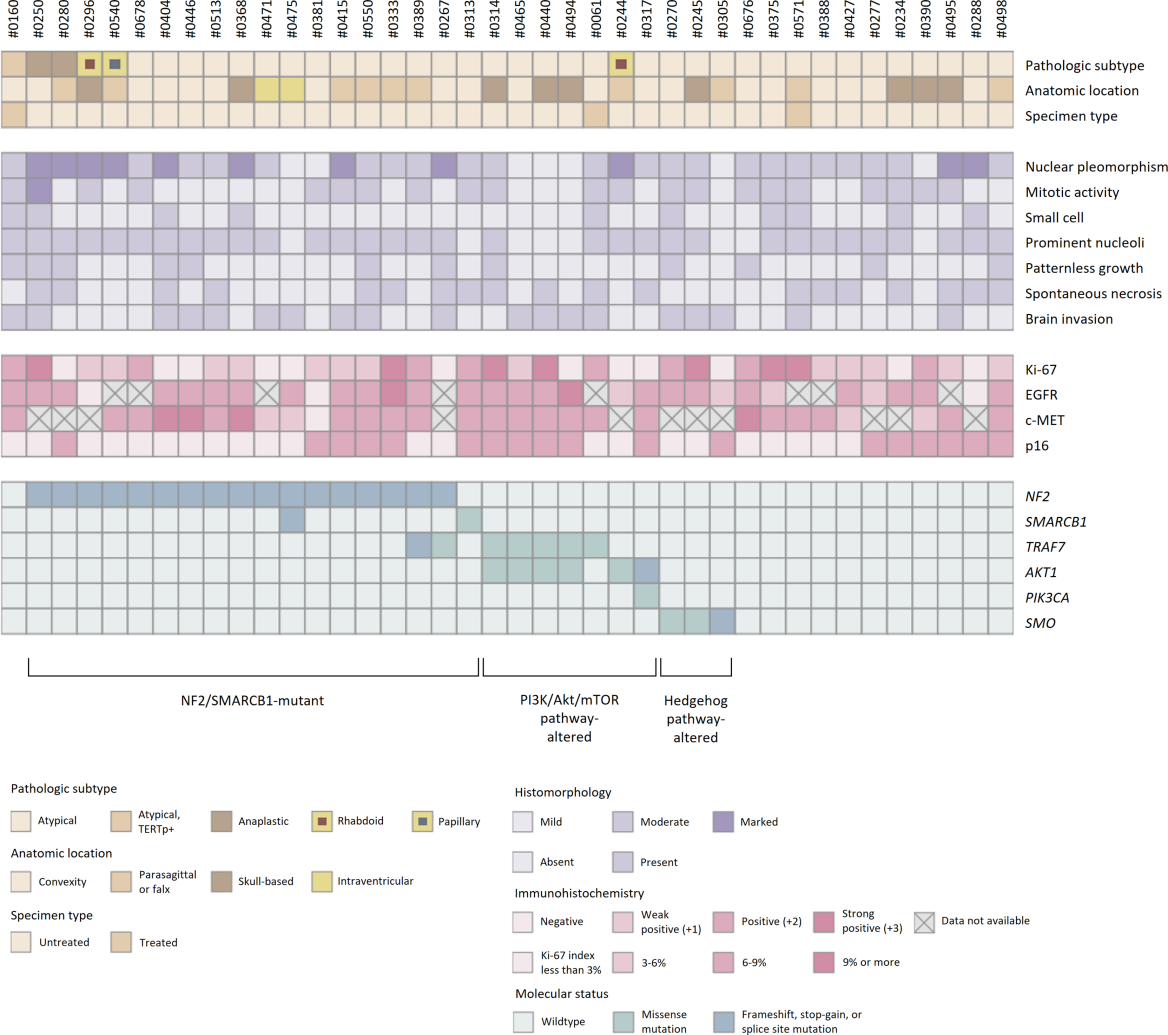
Histopathologic features and molecular status of grade 2/3 meningiomas. Grade 2/3 meningiomas exhibited heterogeneous clinical, histopathologic, and genomic features.

All 40 samples achieved the institutional quality standard of the NGS study. The tumor purity of FFPE samples ranged from 50 to 90%. The sequencing achieved the 100× coverage of 98.0% (96.2-98.5%) for the target regions with a mean depth of 750 (517-901) (**Supplementary Table 2**). A total of 160 somatic variants from 66 different genes were identified (**Supplementary Table 3**). The mutational profiles were highly heterogeneous, and no statistical association between genomic alterations and clinicopathologic features was observed (**Figure 6**).

SNVs and indels were the most frequent in *NF2* (42.5%), which consists of eight frameshift, three stop-gain and five splice site variants. Other mutations in previously documented genes associated with meningiomas, including *AKT1* (one missense and one splice site variants) and *TRAF* (five missense and one splice site variants), were also found. In particular, *AKT1* c.49G>A (p.E17K) mutation was recurrent in five cases, most of which concurrently harbored a missense variant in *TRAF7*. However, there was no clinical relevance in exploratory survival analyses (**Figures 2E, F**).

The prevalence of recurrent variants in meningioma was investigated by comparing the results between pooled data from previous literature and this study (**Table 2**). Interestingly, the prevalence of mutations in *AKT1* and *TRAF7* were significantly higher than that in grade 2/3 cases previously reported in external cohorts [*AKT1* p.E17K mutation in samples vs. external cohorts (#1-6), p < 0.001; *TRAF7* mutations, p = 0.017]. Other known recurrent mutations, such as *KLF4* c.1225A>C (p.K409Q) or *SMO* c.1234C>T (p.L412F), were not detected. A few individual cases presented possible pathogenic variants, *PIK3CA* c.1633G>A (p.E545K) or *SMARCB1* (also known as *BAF47*, *INI1*, or *SNF5L1*) c.1130G>A (p.R377H), while others were tiered as benign or had uncertain significance.

**Table 2:** Prevalence of recurrent variants in meningiomas according to the histopathologic grade.

	External cohort #1	External cohort #2	External cohort #3	External cohort #4	External cohort #5	External cohort #6	Current study	Overall
***NF2* (any variants)**								
Grade 1	28.8% (164 of 570)	36.4% (28 of 77)	22.9% (33 of 144)	23.2% (22 of 99)	36.8% (81 of 220)	38.7% (94 of 243)	.	31.3% (423 of 1353)
Grade 2/3	44.6% (100 of 224)	47.8% (32 of 67)	33.1% (51 of 154)	64.3% (18 of 28)	61.9% (382 of 617)	46.2% (12 of 26)	42.5% (17 of 40)	52.9% (612 of 1156)
***SMARCB1* p.R374Q or p.R377H^†^**								
Grade 1	3.9% (18 of 463)	1.3% (1 of 77)	.	3.0% (3 of 99)	0.9% (2 of 220)	.	.	2.8% (24 of 859)
Grade 2/3	2.1% (4 of 194)	0.0% (0 of 67)	.	3.6% (1 of 28)	0.5% (3 of 617)	.	2.5% (1 of 40)	1.0% (9 of 946)
***TRAF7* (any variants)**								
Grade 1	28.6% (163 of 570)	3.2% (1 of 31)	28.5% (41 of 144)	23.2% (23 of 99)	.	.	.	27.0% (228 of 844)
Grade 2/3	11.2% (25 of 224)	5.3% (3 of 57)	1.3% (2 of 154)	0.0% (0 of 28)	.	.	17.5% (7 of 40)	7.4% (37 of 503)
***AKT1* p.E17K**								
Grade 1	12.6% (72 of 570)	7.8% (6 of 77)	16.0% (23 of 144)	11.1% (11 of 99)	8.6% (19 of 220)	11.1% (27 of 243)	.	11.7% (158 of 1353)
Grade 2/3	6.7% (15 of 224)	4.5% (3 of 67)	0.6% (1 of 154)	0.0% (0 of 28)	2.1% (13 of 617)	3.8% (1 of 26)	12.5% (5 of 40)	3.3% (38 of 1156)
***PIK3CA* p.E542K, p.E545K/A, or p.H1047R/L/Q**								
Grade 1	4.2% (16 of 385)	0.0% (0 of 31)	0.7% (1 of 144)	2.0% (2 of 99)	3.2% (7 of 220)	.	.	3.0% (26 of 879)
Grade 2/3	2.4% (4 of 164)	0.0% (0 of 57)	1.9% (3 of 154)	0.0% (0 of 28)	0.6% (4 of 617)	.	2.5% (1 of 40)	1.1% (12 of 1060)
***KLF4* p.K409Q**								
Grade 1	10.0% (57 of 570)	0.0% (0 of 31)	11.1% (16 of 144)	8.1% (8 of 99)	.	6.6% (16 of 243)	.	8.9% (97 of 1087)
Grade 2/3	2.7% (6 of 224)	0.0% (0 of 57)	0.0% (0 of 154)	0.0% (0 of 28)	.	0.0% (0 of 26)	0.0% (0 of 40)	1.1% (6 of 529)
***SMO* p.L412F or p.W535L**								
Grade 1	4.6% (26 of 570)	3.9% (3 of 77)	3.5% (5 of 144)	4.0% (4 of 99)	2.7% (6 of 220)	0.4% (1 of 243)	.	3.3% (45 of 1353)
Grade 2/3	0.4% (1 of 224)	0.0% (0 of 67)	0.0% (0 of 154)	0.0% (0 of 28)	0.2% (1 of 617)	0.0% (0 of 26)	0.0% (0 of 40)	0.2% (2 of 1156)

Values are percentage (number of cases/total number of screened cases). Data are curated from the following references: External cohort #1 (6, 7); External cohort #2 (8–10); External cohort #3 (11); External cohort #4 (12); External cohort #5 (2); External cohort #6 (13). Variants were screened by either whole genome or exome sequencing, targeted sequencing, Sanger sequencing, or PCR. ^†^Corresponding to R383Q and R386H in GRCh38, respectively.

## Discussion

This study demonstrated the clinical, histopathologic, and genomic characteristics of grade 2/3 meningiomas. In our cohort, there was a significantly larger proportion of males with grade 2/3 meningiomas than with grade 1 meningiomas. This finding was consistent with those observed in a nationwide cohort study and relevant literature (14). Considering a high female predominance in benign meningiomas and the effect of hormones in meningioma pathogenesis (15), it is interesting that sex distribution is more even in higher grades meningiomas. Perhaps, the invasiveness of meningioma might be affected by the different biological factors rather than hormones or other sex-related conditions, which could be discovered from genomic aberrations.

Among the variable morphologic subtypes of meningiomas, our sample population did not show novel findings of recurrent somatic variations. Nevertheless, there were a few interesting points in our results. First, the prevalence of *NF2* mutations in grade 2/3 meningiomas was not as frequent as reported in some previous literature (2, 16). Another known pathogenic gene *SMARCB1*, which has been suggested to be associated with a high Ki-67 index (17), was identified in a single case; however, the histopathologic feature was not specific. Also, we observed that the common recurrent mutations *AKT1* p.E17K and *TRAF7* mutations were as frequent as previously reported in grade 1 meningiomas. Those *AKT1-*mutant and *TRAF7*-mutant cases did not show a significantly different prognosis from the others, which was inconsistent with previous reports (18). Conversely, a recurrent mutation *KLF4* p.K409Q has not been detected in our grade 2/3 samples, which supports that this variant might be predominantly harbored in low-grade tumors and possibly associated with a favorable prognosis without tumor recurrence (17, 19).

The recent article with the updated CNS WHO classification also demonstrated several variants associated with specific subtypes (1). *TERT* promoter mutation, now adopted to molecular diagnosis of grade 3 meningioma, represented highly progressive tumors in our cases. Regarding the emerging issues on molecular diagnosis of meningioma, some experts suggest considering NGS for all grade 2 meningiomas (4): however, there is a lack of consensus on the standardization of diagnostic method regarding the source of specimens, sequencing protocol, or analysis pipeline. Besides, *TERT* promoter mutation was not adequately detectable by targeted NGS study using the common commercial panels, and additional PCR tests have been needed for diagnosis in most institutions. Since the expense of the study is considerably high, the cost-effectiveness of molecular diagnostic studies should be carefully discussed, with full consideration of the patient’s best interest.

Several studies suggested a histopathologic and genomic relevance, but the mutational profiles of meningiomas are highly heterogeneous and inconsistent among previous reports (20). We utilized p16 immunohistochemistry to represent *CDKN2A* deletion; however, it seemed unfeasible to detect the gene-level copy number aberration from protein expression (21). Although some studies also showed certain mutational patterns according to the anatomical location of tumors (18, 22, 23), mutations in *AKT1* or *SMO* that have been frequently reported in skull-based lesions were not specific in our samples. Intraventricular meningiomas, on the other hand, certainly harbored *NF2* mutations without the other common variants, as reported elsewhere (24). Since the clinical significance of most genomic alterations has not been fully understood, further studies are needed to validate the clinicopathologic relevance. Perhaps, different genomic approaches, such as epigenetic studies that were utilized in several studies to identify specific methylation status (11, 25, 26),?A3B2 show [#,32] ?> might provide better understanding of the tumor characteristics.

This study is limited due to the lack of available prognostic measures for patients with meningioma; and as such, the clinical significance of NGS in characterizing the molecular profiles of high-grade meningiomas remains inconclusive. The RFS, the outcome measure utilized in this study, was relatively low compared with other studies (11, 12, 25, 27, 28). Most grade 2/3 meningiomas in this study cohort were successfully controlled by adjunctive treatments, including stereotactic radiosurgery and radiotherapy from conventional decision making without the molecular diagnosis. Future investigations should focus more on specific populations of patients with highly malignant or metastatic lesions that are refractory to the current treatment modalities. In this perspective, molecular targets from NGS can lead to a possible therapeutic implication (15, 29, 30). Nevertheless, for a majority of meningioma cases, surgical outcome and other clinical factors appears to be more critical than tumor properties characterized by NGS at this time.

## Conclusion

Grade 2/3 meningiomas show a broad spectrum of molecular profiles, as they have heterogeneous histologic characteristics. Further studies are needed to validate the clinical significance of the NGS study.

## Data Availability Statement

The datasets presented in this study can be found in online repositories. The names of the repository/repositories and accession number(s) can be found below: ENA, PRJEB51397.

## Ethics Statement

The studies involving human participants were reviewed and approved by the Institutional Review Board in Seoul National University Bundang Hospital. The patients/participants provided their written informed consent to participate in this study. Written informed consent was obtained from the individual(s) for the publication of any potentially identifiable images or data included in this article.

## Author Contributions

All authors contributed to the conception and design of the study. JK performed data analysis and wrote the first draft of the manuscript. KH and KL contributed to material preparation. All authors read and approved the final manuscript.

## Funding

This study was supported by grants from Seoul National University Bundang Hospital (13-2019-0009 & 06-2021-0483).

## Conflict of Interest

The authors declare that the research was conducted in the absence of any commercial or financial relationships that could be construed as a potential conflict of interest.

## Publisher’s Note

All claims expressed in this article are solely those of the authors and do not necessarily represent those of their affiliated organizations, or those of the publisher, the editors and the reviewers. Any product that may be evaluated in this article, or claim that may be made by its manufacturer, is not guaranteed or endorsed by the publisher.
