# Supplementary material for: Clinicopathologic Characteristics of Grade 2/3 Meningiomas: A Perspective on the Role of Next-Generation Sequencing
Source: Front Oncol. 2022 Jun 13;12:885155. doi: 10.3389/fonc.2022.885155 (PMC9236884; doi:10.3389/fonc.2022.885155)
Supplement: Supplementary file 1 [file DataSheet_1.docx]

Supplementary Material

Supplementary Table 1. The complete list of genes that were analyzed in each targeted panel

Supplementary Table 2. Sequencing quality control metrics

Supplementary Table 3. Clinicopathologic information and mutational status of study samples (in a separate data sheet file)

Supplementary Table 1 | The complete list of genes that were analyzed in each targeted panel

| **Brain-specific panel (v1.0)** | | | | | | | | | |
| --- | --- | --- | --- | --- | --- | --- | --- | --- | --- |
| *AKT1*  *ALK*  *ATRX*  *BRAF*  *BRCA1*  *BRCA2* | *CDH1*  *CIC*  *DDX3X*  *EGFR*  *ERBB2*  *FGFR1* | *FGFR2*  *FGFR3*  *FGFR4*  *FUBP1*  *H3F3A*  *HIST1H3B* | *HIST1H3C*  *HRAS*  *IDH1*  *IDH2*  *KIT*  *KLF4* | *KRAS*  *MDM2*  *MDM4*  *MET*  *MLH1*  *MTOR* | *MYB*  *MYC*  *MYCN*  *NF1*  *NF2*  *NOTCH1* | *NOTCH3*  *NRAS*  *NTRK2*  *PDGFRA*  *PDGFRB*  *PIK3CA* | *PTCH1*  *PTCH2*  *PTEN*  *RB1*  *RELA*  *RET* | *ROS1*  *SHH*  *SMARCA2*  *SMARCB1*  *SMO*  *STAT6* | *TERT*  *TP53*  *TRAF7* |
| **Pan-cancer panel (v2.0)** | | | | | | | | | |
| *ABL1*  *ABL2*  *ACVR1*  *ACVR1B*  *ADGRA2*  *AGO2*  *AHNAK2*  *AKT1*  *AKT2*  *AKT3*  *ALK*  *ALOX12B*  *AMER1*  *ANKRO11*  *APC*  *AR*  *ARAF*  *ARFRP1*  *ARID1A*  *ARID1B*  *ARID2*  *ARID5B*  *ASXL1*  *ASXL2*  *ATM*  *ATR*  *ATRX*  *AURKA*  *AURKB*  *AURKC*  *AXIN1*  *AXIN2*  *AXL*  *B2M*  *BABAM1*  *BAP1*  *BARD1*  *BBC3*  *BCL10*  *BCL2*  *BCL2L1*  *BCL2L11*  *BCL2L2*  *BCL6*  *BCOR*  *BCORL1*  *BCR*  *BIRC3*  *BLM*  *BMPR1A*  *BRAF*  *BRCA1*  *BRCA2*  *BRD2*  *BRD3* | *BRD4*  *BRIP1*  *BTG1*  *BTK*  *CALR*  *CARD11*  *CARM1*  *CASP8*  *CBFB*  *CBL*  *CCND1*  *CCND2*  *CCND3*  *CCNE1*  *CD274*  *CD276*  *CD79A*  *CD79B*  *CDC42*  *CDC73*  *CDH1*  *CDK12*  *CDK4*  *CDK6*  *CDK8*  *CDKN1A*  *CDKN1B*  *CDKN2A*  *CDKN2B*  *CDKN2C*  *CEBPA*  *CENPA*  *CHD2*  *CHD4*  *CHEK1*  *CHEK2*  *CIC*  *CREBBP*  *CRKL*  *CRLF2*  *CSDE1*  *CSF1R*  *CSF3R*  *CTCF*  *CTLA4*  *CTNNA1*  *CTNNB1*  *CUL3*  *CXCR4*  *CYLD*  *CYSLTR2*  *DAXX*  *DCUN1D1*  *DDR1*  *DDR2* | *DICER1*  *DIS3*  *DNAJB1*  *DNMT1*  *DNMT3A*  *DNMT3B*  *DOT1L*  *DROSHA*  *DUSP4*  *E2F3*  *EED*  *EGFL7*  *EGFR*  *EIF1AX*  *EIF4A2*  *EIF4E*  *ELF3*  *EMSY*  *EP3DO*  *EPAS1*  *EPCAM*  *EPHA3*  *EPHA5*  *EPHA7*  *EPHB1*  *ERBB2*  *ERBB3*  *ERBB4*  *ERCC2*  *ERCC3*  *ERCC4*  *ERCC5*  *ERF*  *ERG*  *ERRFI1*  *ESR1*  *ETV1*  *ETV4*  *ETV5*  *ETV6*  *EWSR1*  *EZH1*  *EZH2*  *FAM175A*  *FAM46C*  *FAM58A*  *FANCA*  *FANCC*  *FANCD2*  *FANCE*  *FANCF*  *FANCG*  *FANCL*  *FAS*  *FAT1* | *FBXW7*  *FGF10*  *FGF14*  *FGF19*  *FGF23*  *FGF3*  *FGF4*  *FGF6*  *FGFR1*  *FGFR2*  *FGFR3*  *FGFR4*  *FH*  *FLCN*  *FLT1*  *FLT3*  *FLT4*  *FOXA1*  *FOXL2*  *FOXO1*  *FOXP1*  *FRS2*  *FUBP1*  *FYN*  *GABRA6*  *GATA1*  *GATA2*  *GATA3*  *GATA4*  *GATA6*  *GID4*  *GLI1*  *GNA11*  *GNA13*  *GNAQ*  *GNAS*  *GPS2*  *GREM1*  *GRIN2A*  *GRM3*  *GSK3B*  *H3F3A*  *H3F3B*  *H3F3C*  *HDAC9*  *HGF*  *HIST1H1C*  *HIST1H2BD*  *HIST1H3A*  *HIST1H3B*  *HIST1H3C*  *HIST1H3D*  *HIST1H3E*  *HIST1H3F*  *HIST1H3G* | *HIST1H3H*  *HIST1H3I*  *HIST1H3J*  *HIST2H3A*  *HIST2H3D*  *HIST3H3*  *HLA-A*  *HLA-S*  *HNF1A*  *HOXB13*  *HRAS*  *HSD3B1*  *HSP9DAA1*  *ICOSLG*  *ID3*  *IDH1*  *IDH2*  *IFNGR1*  *IGF1*  *IGF1R*  *IGF2*  *IKBKE*  *IKZF1*  *IL10*  *IL7R*  *INHA*  *INHBA*  *INPP4A*  *INPP4B*  *INPPL1*  *INSR*  *IRF2*  *IRF4*  *IRS1*  *IRS2*  *JAK1*  *JAK2*  *JAK3*  *JUN*  *KDM5A*  *KDM5C*  *KDM6A*  *KDR*  *KEAP1*  *KEL*  *KIT*  *KLF4*  *KLHt6*  *KMT2A*  *KMT2B*  *KMT2C*  *KMT2D*  *KMT5A*  *KNSTRN* | *KRAS*  *LATS1*  *LATS2*  *LMO1*  *LRP10*  *LYN*  *LZTR1*  *MAGI2*  *MALT1*  *MAP2K1*  *MAP2K2*  *MAP2K4*  *MAP3K1*  *MAP3K13*  *MAP3K14*  *MAP3K4*  *MAPK1*  *MAPK3*  *MAPK8*  *MAPKAP1*  *MAX*  *MCL1*  *MDC1*  *MDM2*  *MDM4*  *MED12*  *MEF2B*  *MEN1*  *MET*  *MGA*  *MITF*  *MLH1*  *MPL*  *MRE11A*  *MS11*  *MS12*  *MSH2*  *MSH3*  *MSH6*  *MST1*  *MST1R*  *MTOR*  *MUTYH*  *MYB*  *MYC*  *MYCL*  *MYCN*  *MYD88*  *MYOD1*  *NBN*  *NCOA3*  *NCOR1*  *NEGR1*  *NF1* | *NF2*  *NFE2L2*  *NFKBIA*  *NKX2-1*  *NKX3-1*  *NOTCH1*  *NOTCH2*  *NOTCH3*  *NOTCH4*  *NPM1*  *NRAS*  *NSD1*  *NTHL1*  *NTRK1*  *NTRK2*  *NTRK3*  *NUF2*  *NUP93*  *NUTM1*  *PAK1*  *PAK3*  *PAK6*  *PAK7*  *PALB2*  *PARK2*  *PARP1*  *PAX5*  *PAX8*  *PBRM1*  *PDCD1*  *PDCD1LG2*  *PDGFB*  *PDGFRA*  *PDGFRB*  *PDK1*  *PDPK1*  *PGR*  *PHOX2B*  *PIK3C2B*  *PIK3C2G*  *PIK3C3*  *PIK3CA*  *PIK3CB*  *PIK3CD*  *PIK3CG*  *PIK3R1*  *PIK3R2*  *PIK3R3*  *PIM1*  *PLCG2*  *PLK2*  *PMAIP1*  *PMS1*  *PMS2* | *PNRC1*  *POLD1*  *POLE*  *PPARG*  *PPM1D*  *PPP2R1A*  *PPP4R2*  *PPP6C*  *PRDM1*  *PRDM14*  *PREX2*  *PRKAR1A*  *PRKCI*  *PRKD1*  *PRKDC*  *PRSS1*  *PRSS8*  *PTCH1*  *PTEN*  *PTP4A1*  *PTPN11*  *PTPRD*  *PTPRS*  *PTPRT*  *QKI*  *RAB35*  *RAC1*  *RAC2*  *RAD21*  *RAD50*  *RAD51*  *RAD51B*  *RAD51C*  *RAD51D*  *RAD52*  *RAD54L*  *RAF1*  *RANBP2*  *RARA*  *RASA1*  *RB1*  *RBM1O*  *RECQL*  *RECQL4*  *REL*  *RET*  *RFWD2*  *RHEB*  *RHOA*  *RICTOR*  *RIT1*  *RNF43*  *ROS1*  *RPS6KA4* | *RPS6KB2*  *RPTOR*  *RRAGC*  *RRAS*  *RRAS2*  *RSPO1*  *RSPO2*  *RTEL1*  *RUNX1*  *RUNX1T1*  *RXRA*  *RYBP*  *SDHA*  *SDHAF2*  *SDHB*  *SDHC*  *SDHD*  *SESN1*  *SESN2*  *SESN3*  *SETD2*  *SF3B1*  *SH2B3*  *SH2D1A*  *SHOC2*  *SHQ1*  *SLIT2*  *SLX4*  *SMAD2*  *SMAD3*  *SMAD4*  *SMARCA4*  *SMARCB1*  *SMARCD1*  *SMO*  *SMYD3*  *SNCAJP*  *SOCS1*  *SOS1*  *SOX10*  *SOX17*  *SOX2*  *SOX9*  *SPEN*  *SPOP*  *SPRED1*  *SPTA1*  *SRC*  *SRSF2*  *STAG2*  *STAT3*  *STAT4*  *STAT5A*  *STAT5B* | *STK11*  *STK19*  *STK40*  *SUFU*  *SUZ12*  *SYK*  *TAF1*  *TAP1*  *TAP2*  *TBX3*  *TCEB1*  *TCF3*  *TCF7L2*  *TEK*  *TERT*  *TET1*  *TET2*  *TGFBR1*  *TGFBR2*  *TMEM127*  *TMPRSS2*  *TNFAIP3*  *TNFRSF14*  *TOP1*  *TOP2A*  *TP53*  *TP53BP1*  *TP63*  *TRAF2*  *TRAF7*  *TSC1*  *TSC2*  *TSHR*  *U2AF1*  *UPF1*  *VEGFA*  *VHL*  *VTCN1*  *WHSC1*  *WHSC1L1*  *WISP3*  *WT1*  *WWTR1*  *XIAP*  *XPO1*  *XRCC2*  *YAP1*  *YES1*  *ZBTB2*  *ZFHX3*  *ZNF217*  *ZNF703*  *ZNRF3*  *ZRSR2* |

Supplementary Table 2 | Sequencing quality control metrics

|  | **Brain panel, v1.0 (n=37)** | **Pan-cancer panel, v2.0 (n=3)** | **Overall (n=40)** |
| --- | --- | --- | --- |
| **Tumor fraction (%)** | . | 80.0 [75.0, 85.0] | . |
| **Q30 (%)^†^** | 93.0 [92.3, 93.3] | . | . |
| **Total depth (×)** | 1,251 [1,149, 1,384] | . | . |
| **Total reads (10^6^)** | 6.58 [6.27, 7.24] | 38.6 [36.8, 42.3] | . |
| **Target reads (%)** | 64.0 [59.1, 66.9] | . | . |
| **Mean depth (×)** | 749 [515, 905] | 722 [682, 747] | 750 [517, 901] |
| **100× coverage (%)** | 98.0 [96.4, 98.6] | 95.9 [94.9, 96.6] | 98.0 [96.2, 98.5] |
| ≥ 95 | 31 (84) | 2 (67) | 33 (83) |
| ≥ 80 | 6 (16) | 1 (33) | 7 (18) |
| **Uniformity (%)** | 97.0 [96.0, 97.1] | . | . |

*Values are median [IQR] or number (percent). †The percentage of bases having a quality score of 30 or higher (the manufacturer's recommended range: ≥75.0%).*
